# Supplementary material for: Is the BNT162b2 COVID-19 vaccine effective in elderly populations? Results from population data from Bavaria, Germany
Source: PLoS One. 2021 Nov 5;16(11):e0259370. doi: 10.1371/journal.pone.0259370 (PMC8570490; doi:10.1371/journal.pone.0259370)
Supplement: S1 Fig — Vaccine effectiveness was calculated as 1—HR and are adjusted for sex. CI, confidence interval; HR, hazards ratio; VE, vaccine effectiveness. (DOCX) [file pone.0259370.s001.docx]

**S1 Fig. Vaccine effectiveness to prevent COVID-19 infection and related outcomes after at least one BNT162b2 vaccine dose compared to none in Bavarian persons aged 80 years and above.**


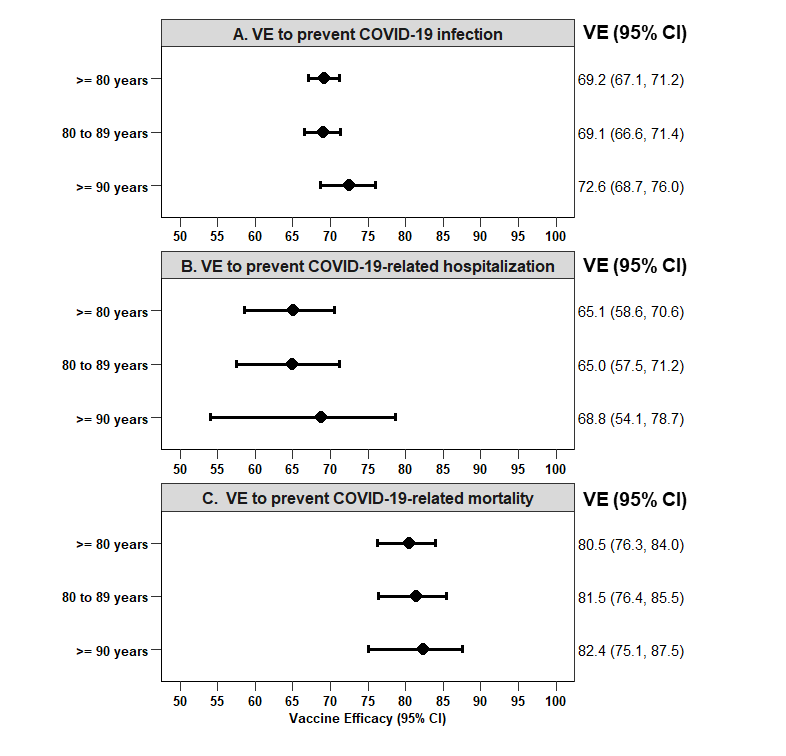
Vaccine effectiveness was calculated as 1 – HR and are adjusted for sex. CI, confidence interval; HR, hazards ratio; VE, vaccine effectiveness.
